# Orthopaedic Surgery Boot Camp: An Immersion Course for Medical Students

**DOI:** 10.7759/cureus.24806

**Published:** 2022-05-07

**Authors:** Yesha Parekh, Paul Romeo, Danika Baskar, Akhil Chandra, Peter Filtes, Bobby Varghese, Tom McPartland, Brian M Katt

**Affiliations:** 1 Department of Orthopaedic Surgery, Rutgers Robert Wood Johnson Medical School, New Brunswick, USA; 2 Pediatric Orthopaedic Surgery, Pediatric Orthopedics Associates, East Brunswick, USA

**Keywords:** orthopaedic surgery sub-internships, fourth-year medical student education, surgical skills-based training, musculoskeletal education, boot camps

## Abstract

Introduction

There is a substantial need for orthopaedic surgery-specific boot camps due to the limited orthopaedic and musculoskeletal education in medical school, which inadequately prepares medical students for their orthopaedic surgery sub-internships. The aim of this study is to identify the impact of the novel orthopaedic surgery boot camp on medical students’ confidence with key orthopaedic topics.

Methods

A cross-sectional study was conducted using an anonymous online survey distributed to medical students attending the novel orthopaedic surgery boot camp. The boot camp consisted of a four-day immersion course into the basics of orthopaedic surgery principles through both didactic and skills-based educational series. The medical students’ confidence in orthopaedic surgery clinical and technical skills were assessed by comparing the students’ survey responses before and after attending each of the sessions.

Results

Twelve fourth-year medical students and 15 second-year medical students attended the boot camp. All the sessions attended by the medical students were statistically significant in improving their confidence in the subject matter and skills-based training. Hundred percent (100%) of the fourth-year medical students recommend future orthopaedic surgery-bound medical students to attend this boot camp.

Conclusion

A dedicated orthopaedic surgery boot camp focused on clinical and technical skills plays a key role in increasing medical students’ confidence with key orthopaedic topics by providing an opportunity to practice these skills in a supervised environment with real-time feedback. This novel boot camp can provide a framework for creating a longitudinal course for medical students to augment the musculoskeletal education taught in medical school education.

## Introduction

Several medical school programs have begun to incorporate a surgical boot camp experience in order to prepare fourth-year medical students for the responsibilities tasked of incoming residents. These immersion courses provide standardized initial exposure to a range of clinical knowledge and technical skills [1]. Effectively executed, these boot camps can increase the confidence and competence of medical students entering their surgical internships [1]. Despite prolonged didactic clinical education, there exists a natural variation of everyday clinical exposure that is not standardized [2]. The curricular didactic education has disproportionately failed to adequately expose students to subspecialty fields they may wish to pursue. The lack of tangible “hands-on” clinical experiences can result in students mainly assuming the role of an observer rather than an active learner [3]. A joint statement released by the American Board of Surgery (ABS), American College of Surgeons (ACS), Association of Program Directors in Surgery (APDS), and Association for Surgical Education (ASE) in 2014 supported the establishment of a surgical preparatory course for students entering surgical residency training programs [4]. The following year, the ACS, APDS, and ASE developed a formal curriculum with specific objectives and goals to guide the creation of educational boot camps for students that have been adopted by several programs across the country [5].

Orthopaedic surgery continues to be one of the most competitive medical residencies for students to match into and necessitates strong performance on objective measures such as United States Medical Licensing Examination® (USMLE®) Step 1 and Step 2 scores, class ranking, and research experiences [6]. Recently, more programs have placed an emphasis on subjective criteria such as performance on clinical rotations and fourth-year sub-internships or “away rotations” [7]. These experiences give program directors insight into applicants’ work ethic, personality, fund of musculoskeletal knowledge, and level of competency with orthopaedic technical skills [7]. Both orthopaedic surgery residency program directors and applicants reported that the value of orthopaedic surgery sub-internships appears to be more utilitarian than educational. Furthermore, both groups state the primary value of the experience is to determine whether the student and residency program appears to be a “good fit” [7]. Therefore, adequate student assessment requires applicants participating in these sub-internships to take a more active role during their sub-internships, rather than just observing. With limited exposure to clinical opportunities in orthopaedic surgery prior to their sub-internships, students may benefit from training on expectations and basic competencies within orthopaedics. Doing so would allow for optimal performance on their fourth-year sub-internships.

It is well-known that orthopaedic and musculoskeletal education continues to be extremely limited and widely variable throughout the preclinical and clinical years of medical school training [8]. Although this further emphasizes the need for dedicated boot camp training programs for trainees pursuing orthopaedic surgery, the literature investigating the implementation of orthopaedic boot camps is deficient, possibly due to the lack of structured pre-residency boot camp equivalents for fourth-year medical students entering the field. The Department of Orthopaedic Surgery at Rutgers Robert Wood Johnson Medical School (RWJMS) has developed a novel orthopaedic surgery boot camp for fourth-year medical students to prepare them for orthopaedic surgery sub-internships. Second-year medical students were also invited to attend parts of the boot camp to provide early exposure to clinical musculoskeletal principles. This course followed a similar set-up as surgical boot camps designed to prepare students for the first year of residency, covering a wide range of didactic lectures and simulation-based training led by the RWJMS Department of Orthopaedic Surgery faculty and orthopaedic residents. We hypothesized fourth-year medical students would demonstrate a significant difference in their self-reported orthopaedic knowledge after completing this educational activity.

This article was previously presented as an abstract at the Medical Student Orthopaedic Symposium on April 10th, 2022.

## Materials and methods

This project was designed as a cross-sectional study using an anonymous online survey that was distributed to medical students at one allopathic medical school who attended an orthopaedic surgery boot camp between June 28, 2021, and July 1, 2021. Medical students who attended the boot camp were queried before and after the sessions. Student responses were not restricted by medical school year. Students who signed up to participate in the boot camp received an anonymous survey on June 27, 2021, which was designed to assess their comfort level with 12 fundamental orthopaedic concepts before beginning this educational activity (Table 1). Students’ self-reported knowledge of orthopaedic topics was scored on a 1.00 to 10.00 Likert scale, with 1.00 being no knowledge of the topic and 10.00 being most knowledgeable for the corresponding topic.

**Table 1:** Orthopaedic Surgery Boot Camp Topics

Orthopaedic anatomy pertaining to common orthopaedic surgical approaches
Orthopaedic implants and modes of internal/external fixation
Using surgical instruments and tissue handling
Fracture and wound healing biology and principles
Interpretation of X-rays
Use of advanced imaging techniques
Assessment of trauma patients/cases
Being a helpful medical student in orthopaedic sub-internship
Applying splints and casts
Suturing and knot tying
Seeing an orthopaedic consult
Conducting an orthopaedic-focused physical examination

The orthopaedic surgery boot camp featured a hybrid of in-person didactic and simulation sessions as well as virtual lectures led by the orthopaedic faculty and residents (Table 2). The duration of these sessions was 60 to 90 minutes. In-person didactic and simulation sessions were designed to provide students with the opportunity to practice orthopaedic skills and receive real-time feedback. The virtual lectures aimed to provide students with not only an introduction to the key orthopaedic topics but also an interactive session for case-based discussions. All didactic and virtual lectures, except for “How to See an Orthopaedic Consult” and “X-Ray Interpretation,” were developed by orthopaedic faculty and residents at our institution. The lectures used for the “How to See an Orthopaedic Consult” and “X-Ray Interpretation,” were developed by Ortho Acting-Intern Coordinated Clinical Education and Surgical Skills (OrthoACCESS) but presented by faculty at our institution [9-10]. OrthoACCESS is a peer-reviewed curriculum designed by orthopaedic educators across the country specifically for fourth-year medical students that focuses on key orthopaedic surgery topics.

**Table 2:** Orthopaedic Surgery Boot Camp Sessions Objectives and Descriptions Rutgers Robert Wood Johnson Medical School (RWJMS); Ortho Acting-Intern Coordinated Clinical Education and Surgical Skills (OrthoACCESS); Magnetic Resonance Imaging (MRI); Longitudinal Relaxation Time (T1); Transverse Relaxation Time (T2); Computed Tomography (CT) [9] Balach T, Curtis D. The Orthopaedic H&P / How to See a Consult. lecture presented at the: OrthoACCESS Webinar #1: The Orthopaedic H&P / How to See a Consult; July 8, 2020. https://cpb-us-w2.wpmucdn.com/voices.uchicago.edu/dist/f/2671/files/2020/07/OrthoACCESS-01-July-Week-1-Orthopaedic-Consults.pdf. Accessed June 1, 2021 [10] Geaney L. X-Ray Interpretation. Lecture presented at the: OrthoACCESS Webinar #2: X-Ray Interpretation; July 15, 2020. https://cpb-us-w2.wpmucdn.com/voices.uchicago.edu/dist/f/2671/files/2020/07/OrthoACCESS-02-July-Week-2-X-Ray-Interpretation.pdf. Accessed June 2, 2021

Day One: Splinting and Casting		
Objectives:	Determine the proper materials needed for casting/splinting a patient. Understand and demonstrate the proper technique for preparing a cast/splint.	
			Location:	Classroom	
Materials:	4-inch soft roll, 4-inch ACE™ Elastic Bandage wrap, 4-inch plaster of Paris casting bandage, 4-inch stockinette, 4-inch fiberglass, bone saw, warm water	
Cost:	$600 (bone saw was borrowed from the orthopaedic surgery clinic)	
Facilitators:	Orthopaedic surgery faculty members and residents	
Description:	Students began by watching a demonstration on how to select the proper materials and prepare the splint/cast. The instruction then detailed the proper placement of a splint and common pitfalls that may be encountered when placing a splint on a patient. Lastly, students were encouraged to demonstrate an understanding of the topic by gathering the necessary materials and splinting on another student.	
Day One: Anatomy Prosection, Dissection, Discussion of Surgical Approaches		
Objectives:	Understand the reasoning behind proper volar and dorsal forearm approaches. Demonstrate proper volar and dorsal forearm approaches.	
Location:	Anatomy/cadaver lab	
Materials:	Cadaver, surgical instruments (scalpels, forceps, retractors, scissors, etc.)	
Cost:	N/A (cadavers and surgical instruments borrowed from the medical school anatomy lab)	
Facilitators:	Orthopaedic surgery faculty members and residents	
Description:	The exercise began with a demonstration of the volar and dorsal surgical approaches to the forearm on a prosected cadaver and how these surgical approaches can be used to ensure key structures (arteries, nerves, and veins) are not damaged. Students were then given the opportunity to practice these approaches in a small group consisting of students and residents.	
			Day One: Review of Orthopaedic Implants and Modes of Internal and External Fixation		
			Objectives:	Learn about different types of internal and external fixation. Determine the principles of when to utilize different devices. Understand the techniques for applying these devices.	
Location:	Classroom				
Materials:	Sawbones and internal and external fixation devices (drills, screwdrivers, plates, intramedullary nails, screws, etc.)	
Cost:	N/A (sawbones and internal and external fixation devices were borrowed from OrthoPediatrics)	
Facilitators:	Orthopaedic surgery faculty members and residents, OrthoPediatrics medical device representatives	
Description:	During this session, students were taught about the basic principles and clinical applications of internal and external fixation devices. Following the discussion, students were provided with the opportunity to learn how to use drills, plates, screws, and intramedullary nails.	
Day One: Social Hour		
Objectives:	Interact with orthopaedic surgery faculty and residents. Learn more about the orthopaedic surgery sub-internships, residency application process, and residency program structure.	
			Location:	Local restaurant	
Materials:	None	
Cost:	$400	
Facilitators:	Orthopaedic surgery faculty members and residents	
Description:	This social hour allowed students to network and seek advice from faculty members and residents prior to starting their fourth-year sub-internships.	
Day Two: Using Surgical Instruments and Tissue Handling		
Objectives:	Learn the name of commonly used surgical instruments. Understand the design and purpose of commonly used surgical instruments. Learn foundational principles of tissue handling and model these techniques on live tissue.	
			Location:	Surgical skills lab	
Materials:	Poultry tissue, surgical instruments (scalpels, forceps, retractors, scissors, sutures, etc.)	
Cost:	$100	
Facilitators:	Orthopaedic surgery faculty member	
Description:	In this session, students learned the names of common surgical instruments and how they enable the surgeon to perform specific surgical techniques. The students were then taught about the principles of tissue handling, including superficial skin incision, dissection of the intermuscular plane, protection of neurovascular bundles, identification of safe anatomical zones, and circumferential periosteal elevation using poultry tissue. The students then practiced these skills on poultry tissue models with a partner and were guided to repair the various tissue planes with appropriate sutures.	
Day Two: Suturing and Knot Tying		
Objectives:	Review commonly used suture methods and materials. Practice suturing on porcine skin models.	
			Location:	Surgical skills lab	
Materials:	Porcine skin, sutures, rope, scalpels, needle drivers, forceps, scissors	
Cost:	$50	
Facilitators:	Orthopaedic surgery faculty member, surgical skills lab coordinator	
Description:	Students reviewed drawings of the following suture types before practicing their techniques on individual porcine skin models: simple interrupted, simple continuous, deep dermal, horizontal mattress, and vertical mattress. Students were also taught the proper method to perform suturing maneuvers in a safe manner.	
Day Two: Physical Examination		
Objectives:	Learn about important components of orthopaedic history taking and physical examination. Practice physical examination maneuvers with another student.	
			Location:	Classroom	
Materials:	None	
Cost:	None	
Facilitators:	Orthopaedic surgery faculty member	
Description:	Students learned how to take a history from patients presenting with musculoskeletal concerns. The faculty member reviewed common physical examination maneuvers at each joint level and demonstrated how to assess the normal range of motion. Students then practiced performing physical exams on a partner.	
Day Two: How to See an Orthopaedic Consult		
Objectives:	Understand the general approach of history-taking and physical examination of a musculoskeletal consult patient in the emergency room or in the clinic. Determine the key pertinent positive and negative findings in HPI and physical examination that should be included in the oral presentation. Describe fractures and degenerative changes on radiographic images. Understand the approach to developing an appropriate assessment and plan as well as differential diagnosis.	
			Location:	Virtual Lecture	
Materials:	Ortho Acting-Intern Coordinated Clinical Education and Surgical Skills (OrthoACCESS) Lecture “The Orthopaedic H&P/How to See a Consult” [9]	
Cost:	None	
Facilitators:	Orthopaedic surgery faculty member	
Description:	The lecture walked through the steps of how to approach a musculoskeletal consult in the emergency room or clinic with regards to history-taking, physical examination, and key radiographic findings. The faculty member presented the three cases in the lecture and had a virtual discussion with the students. Students then practiced taking the history, determined which physical exams they would conduct, read X-rays, and determined the assessment and plan.	
Day Two: Fracture and Wound Healing Biology and Principles		
Objectives:	Describe the difference between cancelous and cortical bone. Describe different types of fractures. Understand the mechanism of fracture and wound healing.	
			Location:	Virtual Lecture	
Materials:	None	
Cost:	Robert Wood Johnson Medical School (RWJMS) Department of Orthopaedic Surgery lecture “Fracture and Wound Healing Biology and Principles”	
Facilitators:	Orthopaedic surgery faculty member	
Description:	This lecture described the anatomy and physiology of bones as well as introduced different types of fractures and their mechanisms of injury. Students were then exposed to the mechanism of fracture and wound healing and how that differs between adults and children.	
Day Three: How to Interpret an X-Ray		
Objectives:	Understand how to read and interpret X-rays of the upper and lower extremities. Describe X-ray findings of osteoarthritis, fractures, and dislocations to colleagues in a standardized manner. Identify the importance of different radiographic views of the upper and lower extremities.	
			Location:	Virtual Lecture	
Materials:	OrthoACCESS lecture “X-Ray Interpretation” [10]	
Cost:	None	
Facilitators:	Orthopaedic surgery faculty member	
Description:	Students learned how to read and interpret X-rays of the upper and lower extremities as well as how to relay the findings to a colleague in a standardized manner. The importance of specific radiographic views of the upper and lower extremity and when they would be indicated were also reviewed during this lecture. The faculty member presented the seven cases in the lecture and had a virtual discussion with the students. Students practiced reading, interpreting, and describing the X-rays to the group.	
Day Three: Understanding Advanced Imaging		
Objectives:	Understand the mechanism of magnetic resonance imaging (MRI). Describe the difference between longitudinal relaxation time (T1) weighted and transverse relaxation time (T2) weighted images. Determine the indications for an MRI compared to an X-ray.	
			Location:	Virtual lecture	
Materials:	RWJMS Department of Orthopaedic Surgery Lecture “Understanding Advanced Imaging”	
Cost:	None	
Facilitators:	Orthopaedic surgery faculty member	
Description:	The lecture described the mechanism behind MRIs and the indications for ordering an MRI compared to an X-ray or computed tomography (CT) image. Students were then exposed to the difference between T1 and T2 images and were given the opportunity to practice reading MRIs.	
Day Four: Assessing a Trauma Patient		
Objectives:	Understand the general approach to assessing a trauma patient. Compare and contrast the management of open and closed fractures. Describe the clinical presentation, pathophysiology, and management of compartment syndrome.	
			Location:	Virtual Lecture	
Materials:	RWJMS Department of Orthopaedic Surgery lecture “Assessing a Trauma Patient”	
Cost:	None	
Facilitators:	Orthopaedic surgery faculty member	
Description:	Students learned about the general approach to assessing a trauma patient as well as the management of open and closed fractures. The lecture also went over the clinical presentation, pathology, and management of compartment syndrome, an orthopaedic emergency.	
Day Four: Being a Helpful Medical Student		
Objectives:	Encourage student participation during sub-internships in the orthopaedic department. Highlight the good qualities and attributes of students who are successful during a sub-internship. Explain the poor qualities and attributes of students that should be avoided when participating in a sub-internship.	
			Location:	Virtual Lecture	
Materials:	RWJMS Department of Orthopaedic Surgery lecture “Being a Helpful Medical Student”	
Cost:	None	
Facilitators:	Orthopaedic surgery second-year residents	
Description:	The “Top 10 Do’s and Do Nots,” when participating in an orthopaedic surgery sub-internship were reviewed during this lecture. The residents were able to share their experiences as sub-interns and answer any questions.	

Following the completion of the boot camp, students received an anonymous survey on July 2, 2021, to reassess their comfort level with the same concepts queried in the pre-boot camp survey. Students were advised to only fill out portions of the post-boot camp survey for which they attended the sessions. Due to concern that survey responses may be linked to future rotating medical students (considering the low sample size in our cohort), the survey was set up as two independent surveys rather than as a paired survey to increase student participation in the project and to protect the integrity of student responses. All responses were collected and managed using Research Electronic Data Capture (REDCap) hosted at RWJMS. REDCap is a secure, web-based software platform designed to support data capture for research studies [11-12]. Rutgers University Health Sciences Institutional Review Board (IRB) - New Brunswick/Piscataway granted IRB approval (Pro2021001581) for this study.

OrthoACCESS internal survey data was utilized for the purposes of comparing this current study’s student responses to a national cohort of students. OrthoACCESS queries medical students regarding their knowledge base on orthopaedic lecture content before and after virtual sessions on a 1.00 to 5.00 scale. OrthoACCESS data were multiplied by a factor of two so that they would be comparable with this current study’s dataset. Fourth-year medical student responses before and after lectures were compared between our project’s dataset and the OrthoACCESS dataset to compare student responses. The same lecture materials were used for all students, only the lecture facilitators varied between the two cohorts.

Fourth-year medical student responses were compared between the pre and post-boot camp surveys utilizing the Mann-Whitney U test. Survey data comparing medical student responses before and after lectures were compared between this current project’s dataset and the OrthoACCESS dataset utilizing the Mann-Whitney U test. Data were reported as “median interquartile range (IQR).” A p-value < 0.05 was considered statistically significant. Analyses were performed using the International Business Machines® (IBM®) Statistical Package for the Social Sciences (SPSS) version 27 (IBM® Corporation, Armonk, NY).

## Results

A total of 12 fourth-year medical students, who were applying for an orthopaedic surgery residency program, participated in the first orthopaedic surgery boot camp facilitated by the RWJMS Orthopaedic Surgery Department. Over the course of the four-day boot camp, the students participated in a total of 12 sessions: six in-person sessions and six virtual sessions. The duration of these sessions was 60 to 90 minutes. One additional session was a social hour amongst orthopaedic surgery faculty, residents, and medical students. Ten out of 12 students attended all sessions, one student only attended the sessions on Day One, and one student only attended the sessions on Days Two to Four. The students were surveyed as to their self-reported level of competency with the key orthopaedic surgery topic before and after attending each of the sessions using a 10-point Likert Scale (1.00 = No knowledge of the topic; 10.00 = Most knowledgeable about the topic) (Table 3).

**Table 3:** Rutgers Robert Wood Johnson Medical School Fourth-Year Medical Student Survey Responses Interquartile Range (IQR) 1.00 = No knowledge of the topic 10.00 = Most knowledgeable about the topic

	Pre-Boot Camp Survey (n=10) Median Interquartile Range [IQR]	Post-Boot Camp Survey (n=11) Median [IQR]	p-value
				Splinting and Casting	4.00 [2.75-5.00]	8.00 [7.00-10.00]	< 0.0001
Anatomy Prosection, Dissection, Discussion of Surgical Approaches	5.00 [2.00-4.00]	7.00 [6.00-9.00]	0.020
Review of Orthopaedic Implants and Modes of Internal and External Fixation	2.50 [2.00-4.00]	7.00 [6.00-8.00]	< 0.0001
Using Surgical Instruments and Tissue Handling	4.00 [3.00-7.25]	9.00 [8.00-10.00]	< 0.0001
Suturing and Knot Tying	6.00 [4.75-7.00]	9.00 [8.00-10.00]	< 0.0001
Physical Examination	3.50 [2.75-4.00]	9.00 [8.00-9.00]	< 0.0001
How to See an Orthopaedic Consult	4.00 [2.75-4.25]	8.00 [8.00-9.00]	< 0.0001
Fracture and Wound Healing Biology and Principles	4.50 [2.75-5.25]	8.00 [8.00-9.00]	< 0.0001
How to Interpret an X-Ray	5.00 [3.00-6.00]	8.00 [8.00-9.00]	< 0.0001
Understanding Advanced Imaging	2.50 [2.00-5.25]	8.00 [7.00-9.00]	< 0.0001
Assessing a Trauma Patient	3.50 [2.00-5.25]	8.00 [7.00-9.00]	< 0.0001
Being a Helpful Medical Student	6.00 [3.50-7.00]	9.00 [8.00-10.00]	< 0.0001

The pre-boot camp survey responses varied from 2.00 to 7.25. Fourth-year medical students prior to the boot camp had a lower self-reported level of competency with the following key orthopaedic surgery topics (median interquartile range (IQR)): “Review of Orthopaedic Implants and Modes of Internal Fixation” (2.50 (2.00 - 4.00)), “Understanding Advanced Imaging” (2.50 (2.00 - 5.25)), “Physical Examination” (3.50 (2.75 - 4.00)), and “Assessing a Trauma Patient” (3.50 (2.00 - 5.25)). The “Suturing and Knot Tying” (6.00 (4.75-7.00)) and “Being a Helpful Medical Student” (6.00 (3.50-7.00)) sessions had a higher level of self-reported competency by fourth-year medical students prior to attending the boot camp sessions.

The post-boot camp survey responses varied from 6.00 to 10.00. All 12 sessions were statistically significant (p < 0.05) in improving fourth-year medical students’ self-reported level of competency when comparing pre-boot camp and post-boot camp survey responses. After attending the boot camp, the four orthopaedic surgery topics that fourth-year medical students had lower self-reported level of competency on the pre-boot camp survey, all showed an increase in their level of competency (median (IQR)): “Review of Orthopaedic Implants and Modes of Internal Fixation” (7.00 (6.00 - 9.00)), “Understanding Advanced Imaging” (8.00 (7.00 - 9.00)), “Physical Examination” (9.00 (8.00 - 9.00)), and “Assessing a Trauma Patient” (8.00 (7.00 - 9.00)). Fourth-year medical students were also asked how beneficial they found the boot camp and if they would recommend the boot camp to future students pursuing a residency in orthopaedic surgery during the post-boot camp survey. In total, 92% of students found the boot camp to be beneficial (scores exceeding 8.00/10.00) in preparing them for their sub-internships and 100% of students would recommend (scores exceeding 8.00/10.00) attending the RWJMS Orthopaedic Surgery Boot Camp to future students interested in pursuing an orthopaedic surgery residency (Table 4).

**Table 4:** Rutgers Robert Wood Johnson Medical School Fourth-Year Medical Students Perception of the Boot Camp 1.00 = Would not recommend 10.00 = Would recommend

Overall, how beneficial did you find the inaugural RWJMS Orthopaedic Education Bootcamp to be?										
Score	1.00	2.00	3.00	4.00	5.00	6.00	7.00	8.00	9.00	10.00
Percent of Total Responses	0.00%	0.00%	0.00%	0.00%	8.33%	0.00%	0.00%	33.33%	25.00%	33.33%
Average Score: 8.67 / 10.00										
How likely are you to recommend the RWJMS Orthopaedic Surgery Boot Camp to future students interested in pursuing an orthopaedic surgery residency?										
Score	1.00	2.00	3.00	4.00	5.00	6.00	7.00	8.00	9.00	10.00
Percent of Total Responses	0.00%	0.00%	0.00%	0.00%	0.00%	0.00%	0.00%	8.33%	8.33%	83.33%
Average Score: 9.75 / 10.00										

The “How to See an Orthopaedic Consult” and “X-Ray Interpretation” virtual lectures as part of the RWJMS Orthopaedic Surgery Boot Camp were developed by Ortho Acting-Intern Coordinated Clinical Education and Surgical Skills (OrthoACCESS) but presented by the RWJMS Department of Orthopaedic Surgery faculty [9-10]. OrthoACCESS also presented these two lectures separately to medical students across the country as part of their lecture series. Both groups of students were surveyed pre and post-session to assess their self-reported competency levels with these specific orthopaedic topics. The OrthoACCESS data (n pre-session survey = 70, n post-session survey = 70) using this 5-point Likert scale (1.00 = Not at all knowledgeable of the topic; 5.00 = Extremely knowledgeable of the topic) was doubled to allow easy comparison with the RWJMS data (n pre-boot camp survey = 10, n post-boot camp survey = 11) that used the 10-point Likert scale (1.00 = No knowledge of the topic; 10.00 = Most knowledgeable of the topic). Statistical analysis comparing the self-reported competency level of RWJMS fourth-year medical students, who attended these sessions as a part of the boot camp, and fourth-year medical students across the country, who attended these sessions as a part of the OrthoACCESS lecture series, was conducted (Table 5).

**Table 5:** RWJMS Fourth-Year Medical Students’ vs OrthoACCESS Fourth-Year Medical Students’ Responses Rutgers Robert Wood Johnson Medical School (RWJMS); Ortho Acting-Intern Coordinated Clinical Education and Surgical Skills (OrthoACCESS) *RWJMS: n pre-boot camp survey = 10, n post-boot camp survey = 11 **OrthoAccess: n pre-session survey =70, n post-session survey = 70 1.00 = No knowledge of the topic 10.00 = Most knowledgeable about the topic

How to See an Orthopaedic Consult	Rutgers Robert Wood Johnson Medical School (RWJMS)* Median Interquartile Range [IQR]	Ortho Acting-Intern Coordinated Clinical Education and Surgical Skills (OrthoACCESS)** Median [IQR]	p-value
Pre-Session Survey	4.00 [2.75-4.25]	6.00 [4.00-6.00]	0.001
Post-Session Survey	8.00 [8.00-9.00]	8.00 [6.00-8.00]	0.006
How to Interpret an X-Ray	RWJMS Median [IQR]	OrthoACCESS Median [IQR]	p-value
Pre-Session Survey	5.00 [3.00-6.00]	4.00 [4.00-6.00]	0.743
Post-Session Survey	8.00 [8.00-9.00]	6.00 [6.00-8.00]	0.001

For the “How to See an Orthopaedic Consult,” session, both RWJMS fourth-year medical students (4.00 [2.75 - 4.25] vs 8.00 [8.00 - 9.00]) and OrthoACCESS fourth-year medical students (6.00 [4.00 - 6.00] vs 8.00 [6.00 - 8.00] reported increased competency with this topic after attending the session. There was a statistically significant difference between the RWJMS and OrthoAccess students pre and post-session (p < 0.05). Similarly, for the “How to Interpret an X-Ray” session, there was an improvement in self-reported knowledge of this topic post-session for both the RWJMS fourth-year medical students (5.00 [3.00 - 6.00] vs 8.00 [8.00 - 9.00]) and the OrthoACCESS fourth-year medical students (4.00 [4.00 - 6.00] vs (6.00 [6.00 - 8.00]). Analysis of the post-session survey between the groups revealed a statistically significant difference between the RWJMS and OrthoACCESS students (p < 0.05).

A total of 15 second-year medical students interested in pursuing a career in orthopaedic surgery also attended the RWJMS Orthopaedic Surgery Boot Camp. These students were invited to attend three of the 12 boot camp sessions. Fifteen of 15 second-year medical students attended the “Splinting and Casting” and “Anatomy Prosection, Dissection, Discussion of Surgical Approaches” sessions, and 13/15 students attended the “Review of Orthopaedic Implants and Modes of Internal and External Fixation” session. The students were surveyed as to their self-reported level of competency with the key orthopaedic surgery topic before and after attending each of these three sessions using a 10-point Likert Scale (1.00 = No knowledge of the topic; 10.00 = Most knowledgeable of the topic) (Table 6).

**Table 6:** Rutgers Robert Wood Johnson Medical School Second-Year Medical Student Survey Responses Interquartile range (IQR) *n pre-boot camp survey = 15, n post-boot camp survey = 13 1.00 = No knowledge of the topic 10.00 = Most knowledgeable about the topic

	Pre-Boot Camp Survey Median Interquartile Range [IQR]	Post-Boot Camp Survey Median [IQR]	p-value
				Splinting and Casting (n=15)	1.00 [1.00-4.00]	9.00 [8.00-10.00]	< 0.0001
Anatomy Prosection, Dissection, Discussion of Surgical Approaches (n=15)	3.00 [1.00-6.00]	8.00 [6.00-10.00]	< 0.0001
Review of Orthopaedic Implants and Modes of Internal and External Fixation (n=15)*	2.00 [1.00-3.00]	8.00 [6.00-9.00]	< 0.0001

The pre-boot camp survey responses varied from 1.00 to 6.00. Second-year medical students prior to the boot camp had the lowest level of self-reported level of competency with the following orthopaedic surgery topic, “Splinting and Casting” (Median (IQR)): 1.00 [1.00 - 4.00]. “Anatomy Prosection, Dissection, Discussion of Surgical Approaches” (3.00 [1.00 - 6.00]) had the highest level of self-reported competency by second-year medical students prior to attending the boot camp sessions. After attending these three sessions, the second-year medical students were surveyed again to evaluate if the boot camp sessions improved their self-reported level of competency with the key orthopaedic surgery topics. The post-boot camp survey responses varied from 6.00 to 10.00. All three sessions were statistically significant (p < 0.0001) in improving second-year medical students’ self-reported level of competency when comparing pre-boot camp and post-boot camp survey responses.

## Discussion

The main forces driving the development of boot camps evolved from a concern that incoming surgical interns are not adequately prepared for the challenges of patient care and that the fourth year of medical education can be improved [1]. These boot camps have shown an increase in the confidence and competence of medical students' abilities to perform clinical and technical skills prior to their surgical internships [1]. One study demonstrated an increase in skill performance equivalent to six months of internship after the completion of a two-and-a-half-day boot camp, which included varied skill techniques such as chest tube placement, central line placement, and cricothyroidotomy [13]. Boot camps consisting of lecture and didactic instruction for medical students have shown significant increases in the self-reported confidence of students [14]. Self-reported improvements have been seen in categories such as preparedness for sub-internship, sewing, opening/closing principles, and system-focused examinations [14]. High student satisfaction has also been ascertained over the course of boot camps aimed at teaching skills essential in surgery such as those related to both basic surgical technical skills as well as advanced laparoscopic or robot-assisted surgical skills, with 78% of the 45 participating students in a boot camp every year were “very satisfied'' while 22% reported being “quite satisfied” [15]. This demonstrates the importance of boot camps as a beneficial part of medical education to adequately prepare medical students to take on the responsibilities of an incoming resident.

Further research has also been conducted on the facilitation of these boot camps. One study examining the effectiveness of a boot camp designed for first-year orthopaedic surgery residents involved didactic and simulation-based training led by orthopaedic surgery surgeons and senior residents and followed by hands-on attendee practice [16]. The didactic lectures covered a wide range of topics, including surgical simulation and cadaver dissection, soft-tissue handling, basic fracture fixation, casting and splinting, as well as familiarization with basic surgical instruments [16]. This study found a significant difference in procedural performance among the group that underwent a 30-day intensive skills course, including cadaver dissection, surgical simulation, soft-tissue handling, casting and splinting, basic fracture fixation, and surgical instrument familiarization when compared to residents who did not undergo a boot camp experience [16]. These results support the establishment of early boot camp training programs for orthopaedic surgery trainees. Our study results are consistent with the literature, as our fourth-year medical students who participated in the intensive orthopaedic boot camp showed a significant increase in their self-reported knowledge of orthopaedic skills, specifically orthopaedic implants and internal fixation, advanced imaging, physical examination, and assessing trauma patients after attending the educational sessions.

Though it has been previously acknowledged that modern medical education lacks sufficient training in musculoskeletal principles, little has been done to address this deficit [8,17-18]. Building an early foundation becomes even more imperative for those pursuing a career in orthopaedic surgery, as the management of musculoskeletal trauma and disorders forms the cornerstone of their training and practice. This further emphasizes the need for a tailored curriculum that provides an overview of musculoskeletal concepts and related surgical skills, all of which can be achieved through a structured orthopaedic immersion course. While this may best serve fourth-year medical students who will be rotating on an orthopaedic service, our results show that offering this training even earlier can be beneficial. Second-year medical students who attended some sessions of our boot camp showed a significant increase in self-reported competence with splinting and casting, surgical anatomical concepts, and orthopaedic implant and fixation knowledge. This demonstrates that early orthopaedic skills and knowledge training may help to build confidence with an overall understanding of these key topics. The boot camp helped develop a foundation of knowledge that can be expanded upon and reinforced through clinical rotations.

Given the limited sample size of the Rutgers Robert Wood Johnson Medical School (RWJMS) fourth-year medical students (n pre-boot camp survey = 10, n post-boot camp survey = 11), the RWJMS dataset was compared with the Ortho Acting-Intern Coordinated Clinical Education and Surgical Skills (OrthoACCESS) dataset (n pre-boot camp survey = 70, n post-boot camp survey = 70), a cohort that is representative of fourth-year medical students across the country, for the “How to See an Orthopaedic Consult” and “X-Ray Interpretation” virtual lectures. There was a statistically significant difference (p < 0.05) between the RWJMS students and OrthoACCESS students with regard to their self-reported level of competency after attending both these lectures. Both groups showed an improvement in their self-reported competency with these two lectures when comparing pre and post-session survey responses. This demonstrates that these virtual lectures can improve fourth-year medical students’ knowledge about seeing orthopaedic consults and interpreting X-rays when taught in an educational lecture series. Further investigation with a larger cohort is needed to adequately compare the self-reported level of competency in key orthopaedic surgery between RWJMS fourth-year medical students and OrthoACCESS fourth-year medical students.

During the post-boot camp survey, fourth-year medical students were also given an option to provide written feedback on both the in-person and virtual sessions as well as suggestions on how to improve the boot camp for the subsequent years. The fourth-year medical students especially enjoyed the hands-on skills sessions: “Splinting and Casting,” “Anatomy Prosection, Dissection, Discussion of Surgical Approaches,” “Using Surgical Instruments and Tissue Handling,” and “Suturing and Knot Tying.” They found these sessions to be helpful in providing an introduction and opportunity to practice these technical skills in a safe and supervised environment prior to attending their sub-internships, especially since many of the skills specific to orthopaedic surgery are not taught or stressed in medical school education. Medical students generally are only exposed to these orthopaedic-specific skills during their orthopaedic surgery electives or sub-internships. Fourth-year medical students found the “Physical Examination” and “How to Interpret an X-Ray” sessions to be the most helpful to developing clinical skills since there is also little exposure to musculoskeletal physical examination techniques and X-ray interpretations in medical school. In addition, these students also enjoyed the “Social Hour” because it provided a way for the students to get to know each other as well as the faculty and residents and ask each other questions in preparation for their orthopaedic surgery sub-internships and residency applications.

Second-year medical students were also given the opportunity to provide written feedback through the post-boot camp survey for the three in-person sessions they attended as part of the boot camp. They enjoyed these interactive sessions and thought they served as a great introduction to orthopaedic and musculoskeletal education. Many students wrote they wanted to attend the rest of the sessions reserved for the fourth-year medical students and are looking forward to attending the complete boot camp as fourth-year medical students.

Based off of the constructive feedback provided by the second and fourth-year medical students, the RWJMS Orthopaedic Surgery Boot Camp can be improved for subsequent years by incorporating the following suggestions: encouraging more orthopaedic faculty and resident involvement to increase the exposure of students to the entire department prior to their sub-internships, disseminating pre-recorded podcasts and objectives for review prior to the sessions, and including more case-based discussions in the virtual lectures. Students also mentioned covering these clinical activities and lectures over the course of the year as a longitudinal educational series, having a smaller student to faculty/resident ratio in the technical skills sessions to allow more personalized attention, and separating the fourth-year medical students from the second-year medical students would allow for the provision of orthopaedic education at a knowledge-base appropriate for each level of medical school education.

There are a few limitations to our study. Since not all fourth-year medical students attended every session of the boot camp, our data are missing some post-boot camp survey responses. Our study also had a low number of participants and is likely not adequately powered to detect a beta error. For these reasons, we sought additional data points from OrthoACCESS to strengthen our number of responses. The OrthoACCESS surveys were scaled from 1.00 to 5.00, as opposed to our survey, which was scaled from 1.00 to 10.00. Therefore, to adequately conduct statistical analysis, the responses had to be multiplied by a factor of two. A further limitation of this study design was the need to protect our respondents' anonymity. Due to the limited number of students attending the boot camp sessions and the future inclusion of these students on orthopaedic rotations within our institution, the survey was created as two separate surveys, which were then compared as independent samples. While this limits the data due to a lack of paired comparisons, we believe it augments the integrity of our students’ responses while protecting their identities. A future direction to evaluate the effectiveness of this boot camp in preparing fourth-year medical students for their sub-internships would be for orthopaedic surgery faculty to formally assess fourth-year medical students’ skills before and after the boot camp as well as during the sub-internship.

## Conclusions

As applying for an orthopaedic surgery residency training program after medical school becomes increasingly competitive, there is an increasing need for orthopaedic surgery-specific boot camps. These boot camps serve to fill the gap in fourth-year musculoskeletal and orthopaedic knowledge while also providing them with the clinical and technical skills that will allow for optimal performance on their sub-internships, which play an increasingly important role in a successful match into an orthopaedic surgery residency program. The RWJMS Orthopaedic Surgery Boot Camp serves as a model that medical schools across the country can adopt to create educational opportunities for medical students interested in pursuing an orthopaedic surgery residency. All sessions attended by the fourth-year medical students demonstrated an increase in their confidence with the key orthopaedic surgery clinical and technical skills. They also found the boot camp beneficial in preparing them for their orthopaedic surgery sub-internship, which shows the importance of conducting orthopaedic surgery-specific boot camps for fourth-year medical students before they attend their sub-internships. By including second-year medical students as part of the boot camp, our study has shown that students at this level are eager to receive more orthopaedic-specific clinical skills and didactic sessions and emphasizes the need for more longitudinal orthopaedic and musculoskeletal educational opportunities for medical students of all levels of medical education. Orthopaedic surgery-specific boot camps are crucial to adequately prepare medical students with the confidence, knowledge, and clinical and technical skills necessary to excel during their orthopaedic surgery sub-internships, an important aspect of the orthopaedic surgery residency program application.
